# Rate of viral load failure over time in people on ART in the UK Collaborative HIV Cohort (CHIC) study

**DOI:** 10.7448/IAS.17.4.19527

**Published:** 2014-11-02

**Authors:** Jemma O'Connor, Colette Smith, Fiona Lampe, Margaret Johnson, Caroline Sabin, Andrew Phillips

**Affiliations:** 1 Department of Infection and Population Health, UCL, London, UK; 2 Ian Charleson Day Centre, Royal Free Hampstead NHS Trust, London, UK

## Abstract

**Introduction:**

Most people achieve and maintain viral load (VL) suppression on first-line antiretroviral therapy (ART) but for a minority this does not happen. It is unclear whether those who have maintained VL suppression for several years will be able to continue to do so, or if rates of VL failure – due to poor adherence, ART interruption and/or resistance – remain at appreciable levels.

**Methods:**

Eligible participants were ART-naïve and started treatment after 1st January 2000, with ≥3 antiretrovirals and ≥9 months follow-up. VL failure was defined as failure to achieve VL suppression (≤50 copies/mL) by 9 months on ART or a single VL >200 copies/mL after 9 months after start of ART. Kaplan-Meier estimates were used to examine the cumulative probability of experiencing a VL >200 copies/mL over time, irrespective of treatment interruption (Figure 1). Follow-up was censored at last VL assessment but not at treatment interruption. In a sensitivity analysis, VL failure was instead defined as two consecutive VL >1000 copies/mL.

**Results:**

A total of 13,556 participants were included. Median (IQR) age at start of ART was 37 (32–43), median follow-up 4.1 (2.3–6.7) years, pre-ART VL 71,400 (17,400–221,900) copies/mL and pre-ART CD4 count 204 (110–290) cells/mm^3^. Fifty-one percent were white, 71% male and 50% MSM. Of which, 5,351 (39%) participants experienced a VL >200 copies/mL. In sub-groups of participants the proportion experiencing a VL >200 copies/mL by one year after start of ART were: <50 years 22%, ≥50 years 17%, men 20%, women 26%, MSM 19%, black heterosexuals 23%, white heterosexuals 26% and other 23%. The median time to VL >200 copies/ml was 8 years. In sensitivity analyses based on 12,811 participants, 4274 (33%) experienced two consecutive VL >1000 copies/mL. Table 1 presents the rate of experiencing a VL >200 copies/mL (two consecutive VL >1000 copies/mL) by time since start of ART. The rate of VL >200 copies/mL declines over time, from 30 per 100 person-years after up to two years after ART, to two per 100 person-years after up to 11.5 years after ART. A sum of 2,047 (15%) participants stopped ART at some point (10, 14 and 17% had stopped ART by 1, 3, and 5 years, respectively).

**Conclusions:**

Although resistance will often not be present and, even if present, several drug options will likely remain, first occurrence of VL > 200 copies/mL after having attained viral suppression continues to occur after 10 years on ART.

**Figure 1:**
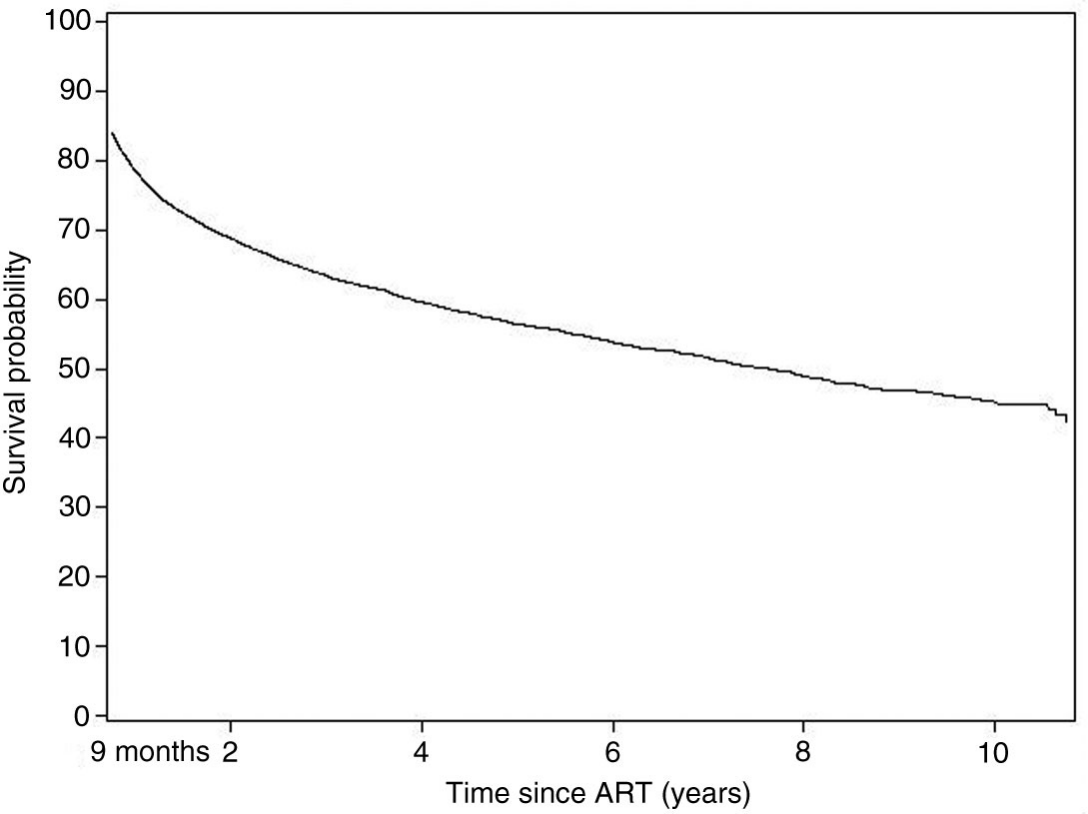
Kaplan-Meier plot of time to viral load failure (single VL > 200 copies/mL) **since initiation of ART**.

**Table 1:** Rate per 100 person-years of viral load failure over time since start of ART

		Single VL >200 copies/mL (two consecutive VL >1000 copies/mL)		

Follow-up on ART, years (x)	Number in risk set at start of period	Number of VL failures during period	Rate of VL failure per 100 person-years	95% confidence interval for rate of VL failure
0x ≤ 2	13,556 (12,811)	4,075 (3,376)	30.1 (26.4)	29.2–30.8 (25.6–27.1)
2x ≤ 4	7,310 (7,710)	801 (577)	11.0 (7.5)	10.3–11.7 (6.9–8.1)
4x ≤ 6	3,992 (4,434)	305 (204)	7.6 (4.6)	7.2–8.8 (4.0–5.2)
6x ≤ 8	2,085 (2,426)	128 (89)	6.1 (3.7)	5.0–7.0 (2.9–4.4)
8x ≤ 10	805 (980)	38 (26)	4.7 (2.7)	3.5–6.5 (1.6–3.7)
10x ≤ 11.5	179 (220)	4 (2)	2.2 (0.9)	0.4–3.8 (0.1–2.4)

